# Vitamin A Supplementation at Birth Might Prime the Response to Subsequent Vitamin A Supplements in Girls. Three Year Follow-Up of a Randomized Trial

**DOI:** 10.1371/journal.pone.0023265

**Published:** 2011-08-11

**Authors:** Ane Bærent Fisker, Peter Aaby, Amabelia Rodrigues, Morten Frydenberg, Bo Martin Bibby, Christine Stabell Benn

**Affiliations:** 1 Bandim Health Project, Indepth Network, Bissau, Guinea-Bissau; 2 Bandim Health Project, Statens Serum Institut, Copenhagen S, Denmark; 3 Department of Biostatistics, Institute of Public Health, University of Aarhus, Aarhus, Denmark; The University of Adelaide, Australia

## Abstract

**Objectives:**

Within a randomised trial of neonatal vitamin A supplementation (VAS) in Guinea-Bissau, neonatal VAS did not affect overall infant mortality. We conducted a post-hoc analysis to test the hypothesis that neonatal VAS primes the response to subsequent vitamin A.

**Methods:**

All trial children were offered VAS after follow-up ended at 1 year of age (FU-VAS). We compared mortality between 1 and 3 years of age according to initial randomization to neonatal VAS or placebo in Cox-regression models; we expected that children randomized to neonatal VAS compared with those randomized to placebo would have lower mortality after reception of FU-VAS.

**Results:**

Of 4345 infants enrolled in the original trial, 3646 lived in the study area at 1 year of age and 2958 received FU-VAS. Between 1 and 3 years of age, 112 children died. After FU-VAS, neonatal VAS was associated with lower mortality than placebo: Mortality Rate Ratio (MRR) = 0.54 (95%CI: 0.31–0.94). The effect was more pronounced in girls (MRR = 0.37 (0.16–0.89)) than boys (MRR = 0.73 (0.35–1.51)). The beneficial effect of neonatal VAS may have been particularly strong for girls who received both VAS in a campaign and FU-VAS (MRR = 0.15 (0.03–0.67)). Among children who had not received FU-VAS, mortality in the second and third year of life did not differ according to reception of neonatal VAS or placebo. Hence, in the second and third year of life the effect of neonatal VAS versus placebo was different in girls who had or had not received FU-VAS (p for homogeneity = 0.01).

**Conclusions:**

The present results suggest that neonatal VAS primes the response in girls such that they get a beneficial effect after a subsequent dose of VAS.

**Trial Registration:**

Clinicaltrials.gov NCT00168597

## Introduction

Meta-analyses have estimated that vitamin A supplementation (VAS) after 6 months of age reduces all-cause mortality by 23–30% in low-income countries [1], [2]. The WHO therefore recommends VAS at vaccination contacts after 6 months of age and at national immunization days when routine coverage with VAS is less than 80% [3]. Hence, children may receive VAS on several occasions. The original VAS trials provided several doses of VAS with 4- or 6-monthly [4]–[8] intervals. Three trials presented the mortality reduction by dose [7]–[9] and in two of the trials, the beneficial effect of vitamin A seemed most pronounced after a second dose [8], [9].

In a randomized trial comparing two different doses of VAS given with oral polio vaccines (OPV) during national immunization days in Guinea-Bissau, a low dose was better for girls, whereas the dose difference mattered little for boys [10]. When we repeated the trial, more children had received VAS previously, and the high dose of VAS tended to be more beneficial in these children (submitted paper). This made us hypothesize that a first dose of VAS may prime a more beneficial response to subsequent VAS.

Between 2002 and 2005, we conducted a randomized trial of VAS provided with Bacille Calmette-Guérin vaccine (BCG) at birth to study the effect on infant mortality [11]. We found no overall effect of VAS, the mortality rate ratio for VAS versus placebo at birth being 1.07 (0.79–1.44) [11]. All children were offered VAS (100,000 IU) at a follow-up visit after 1 year of age. This provided an opportunity to study the hypothesis that neonatal VAS primed the response to subsequent VAS, leading to improved survival in children who received VAS at birth and at follow-up.

## Methods

### Setting

The Bandim Health Project (BHP) keeps a health and demographic surveillance system covering approximately 100,000 inhabitants in six suburban districts of the capital of Guinea-Bissau. All children in the study area are followed with home visits every three months until the age of three years to register vaccinations, hospitalizations, and survival.

Guinea-Bissau is classified as having sub-clinical vitamin A deficiency by UNICEF [12]. Within the present cohort 27% had low retinol binding protein (corresponding to <0.70 µM retinol) at 6 weeks and 9% at 4 months of age, controlled for acute infection [13]. Vitamin A campaigns for children aged 6 months to 5 years were conducted in November 2003, November 2004, November 2005, and in May and November 2006. The BHP registered all children in the study area who received VAS during these campaigns. Within the VAS-at-birth trial we found at 12 months of age that 23% of the children were stunted and 8% underweight (length- and weight-for-age z-score <−2) [14].

### Enrolment

The VAS-at-birth trial has been described in details elsewhere [11]. Mothers of normal birth weight infants (> = 2500 g) without signs of overt illness or malformations were invited to participate when the child was due to be BCG-vaccinated, either shortly after giving birth at the maternity wards, or when the mother brought the child for BCG vaccination at the health centers after a home delivery. Mothers were informed by a trained field worker about the study and asked if they wanted their child to participate. Since the majority of the mothers are illiterate verbal consent was obtained (specified in the approved protocol). Written consent was obtained if the mother knew how to write her own name. If not, a statement that she had understood the information given and agreed to participate was signed by the assistant. Provided consent, the mother drew a lot from an envelope prepared by the study supervisor. Each envelope contained 100 lots: 50 marked “1” and 50 marked “2”, indicating from which of two numbered bottles, “1” or “2,” the child should receive the supplement. The lots were folded, making it impossible to tell what was written on them before they were opened. A new envelope was not taken into use before the previous envelope had been completely emptied. VAS was ½ ml of oil containing 50,000 IU vitamin A as retinyl palmitate and 10 IU vitamin E and placebo was ½ ml of oil containing only 10 IU vitamin E. The code was broken 12 months after the last child was enrolled. We found no adverse effects of 50,000 IU vitamin A with BCG vaccine at birth [15].

### VAS at follow-up visits

All trial children were visited at home by a special team after turning 1 year of age. Repeated visits were made to children who were absent or travelling. Children who had moved within the urban study area were localized if possible and visited at the new address. Field workers conducting the 1-year visit were unaware of the treatment allocation at enrolment. At the 1-year visit an interview on previous VAS doses and hospitalizations was conducted and the child was offered 100,000 IU vitamin A (FU-VAS) if it had not received campaign VAS in the preceding month. The dose of 100,000 IU rather than 200,000 IU was used because previous results had indicated that the lower dose was associated with better survival [10]. The initiation of the 1-year visits was delayed for logistical reasons. Hence, children enrolled in the first year had later follow-up visits than children enrolled later.

All children were followed for mortality through the demographic surveillance system until the age of 3 years. Deaths were registered at the 3-monthly home visits. The registration of a death is followed by a short interview about the cause of death conducted by a local physician or midwife.

### Generation of the “priming hypothesis”

We did not plan to study how neonatal VAS affects the response to a later dose of VAS when we designed the trial of neonatal VAS. However, contrasting effects of two previous randomized trials comparing the recommended dose of VAS and half this dose conducted during VAS campaigns in 2002 and 2004, made us hypothesize that the effect of VAS depends on prior dosing: In the 2002-trial, the low dose (half of the recommended 100,000/200,000 IU for children 6–11/>12 months respectively) was more beneficial than the high dose for girls [10]. In the 2004-trial, conducted to test this observation, there was no beneficial effect of the low dose (submitted). More children had received VAS previously in the 2004-trial, and children who had received VAS previously tended to benefit from the high dose. We therefore hypothesized that the effect of VAS may depend on prior supplementation. Since the neonatal VAS trial had randomized the children to VAS or placebo at enrolment and had provided a follow-up dose of VAS (FU-VAS) to all participants, we used the data set to test whether mortality after FU-VAS depended on the prior randomization to VAS or placebo. The longitudinal registration of undertaken by the BHP made this study possible in spite of not being planned.

### Testing the “priming hypothesis”

**Figure 1:**
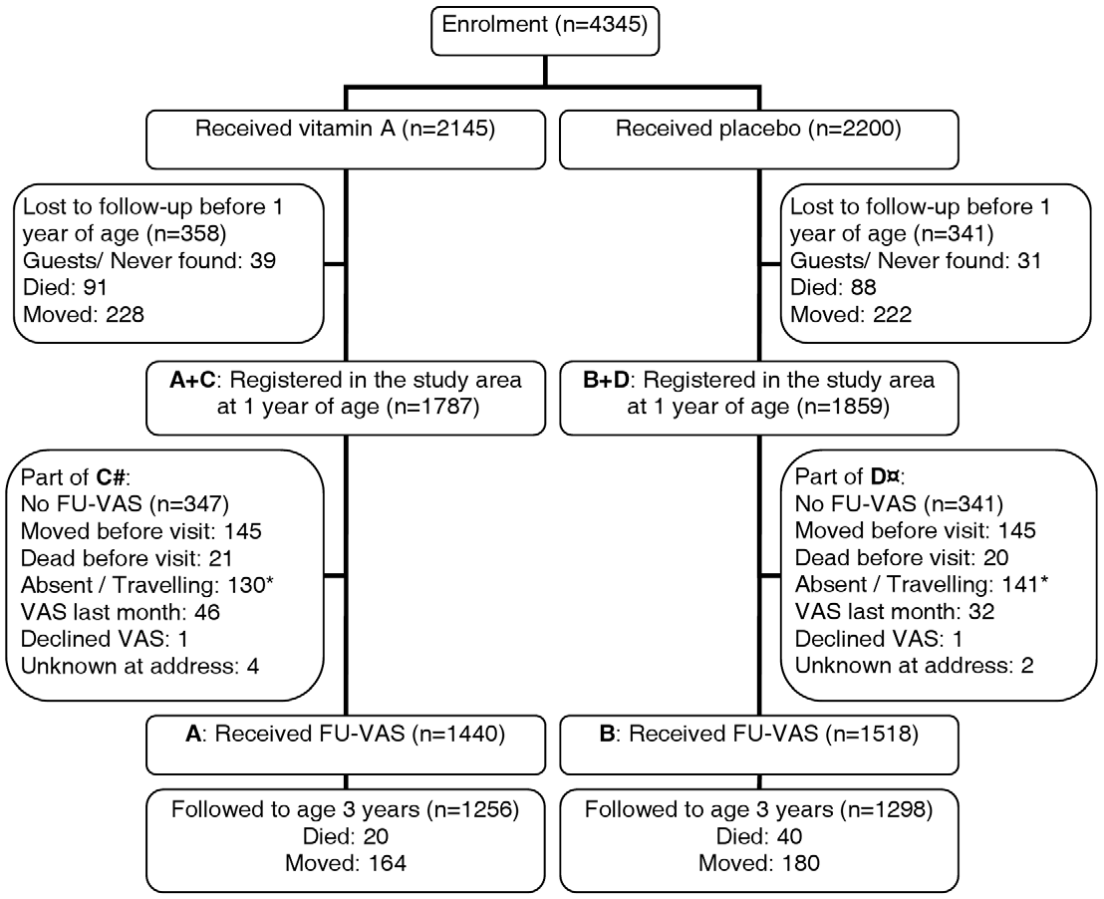
Trial profile. # Note: children in “A” contribute survival time in group C between age 12 months and date of reception of FU-VAS. ¤ Note: children in “B” contribute survival time in group D between age 12 months and date of reception of FU-VAS. * Among absent and travelling children 11 deaths occurred before 3 years of age (Vitamin A: 6; placebo: 5), bringing the total number of deaths up to 112.

Had the study been planned, we would have randomized to VAS or placebo at birth (as done) and randomized the children again at 12 months of age to FU-VAS or placebo (not done) to get groups A, B, C and D (Figure 1). In this prospective follow-up study, our groups A and B consist of children who were met at home at the follow-up visit and received FU-VAS before 3 years of age. Children still living in the study area at 12 months of age, who had not yet received FU-VAS formed groups C and D; some of these children never received FU-VAS and some received FU-VAS later and then changed to groups A and B.

To examine whether VAS-at-birth primed the response to FU-VAS, we compared survival of children who had received FU-VAS according to randomization to VAS or placebo at birth, i.e. comparing groups A and B (trial profile, Figure 1). For priming to be important group A should have lower mortality than group B even though both groups had received VAS after one year of age. Additionally, we investigated whether the lower mortality of A than B was due to the initial profiling of the immune system by VAS by comparing the effect of VAS versus placebo at birth both during the first year of life and during the second/third year of life among those who had not received FU-VAS (groups C and D).

### Ethics Statement

The protocol for the neonatal VAS trial was approved by the Ministry of Health in Guinea-Bissau (‘Núcleo de Coordenação de Pesquisa, Ministerio da Saude Publica’) and ‘The Danish National Committee on Biomedical Ethics’ gave its consultative approval. The original trial was registered at clinicaltrials.gov, number NCT00168597.

### Statistical methods

Survival was assessed in Cox proportional hazards models with age as underlying time, comparing the mortality rates in the two groups originally randomized to VAS or placebo at birth. Hence, to compare the two groups we provided mortality rate ratios (MRR) controlled for age. Follow-up time was censored at 3 years of age, or date of moving out of the area, whichever came first. Accidental deaths were censored at the date of death.

Our primary analysis was an analysis comparing those randomized to VAS or placebo at birth after reception of FU-VAS (groups A and B). In the secondary analyses we compared the effect of VAS versus placebo at birth both during the first year of life and during the second/third year of life among those who had not received FU-VAS (groups C and D). Furthermore, we studied whether additional VAS in campaigns before FU-VAS modified the effect of neonatal VAS versus placebo. We used FU-VAS rather than VAS in campaigns in our primary analysis of priming since the information on FU-VAS was available for all enrolled children. Since the children were followed to 3 years of age, we also assessed the long-term effect of neonatal VAS between birth and 3 years of age regardless of subsequent doses of VAS. Hence, all comparisons were based on the comparison of the two groups of children who were originally randomized to neonatal VAS or placebo.

All analyses were stratified by sex as VAS may have sex-differential effects [10], [16]; estimates are reported for boys and girls separately as well as combined.

Sub-group analyses examined whether the effect of neonatal VAS was limited to certain sub-groups; we stratified by nutritional status at FU-VAS, timing of follow-up, maternal education, and place of enrolment. Effect modification was analyzed by investigating the homogeneity of the effect of neonatal VAS in the different categories of the suspected modifier using Wald test statistics.

The proportional hazard assumption was assessed using Schoenfeldt's residuals and by visual inspection of log-log plots. Visual inspection of log-log plots suggested that the proportional Hazards assumption was violated the first months after FU-VAS, though this was not confirmed by Schoenfeldt's residuals. We tested whether the estimate changed, if we excluded the first 2 months after FU-VAS (during which 12 deaths occurred). This was not the case and all results have therefore been presented for the whole period. All analyses were conducted using Stata 10.1.

## Results

### Study population

The study profile is presented in Figure 1. Between November 13, 2002, and November 28, 2004, we enrolled 4345 children of whom 3646 (all: 84%; vitamin A: 87%, Placebo: 81%) were still living in the study area at 12 months of age. Of these, 2958 (vitamin A: 81%; placebo: 82%) were met at home at the follow-up visit and received FU-VAS before 3 years of age (groups A and B). Among those still living in the study area at 12 months of age, 688 never received FU-VAS and formed groups C and D along with children who had not yet received FU-VAS. Children were followed to 3 years of age, thus the follow-up ended November 28, 2007. We registered a total of 112 deaths (vitamin A at birth: 47, placebo at birth: 65) in the second and third year of life. Based on simple death interviews 108 deaths were due to infectious disease, whereas 4 deaths were accidents and censored at the date of death (vitamin A: 1 (intoxication), placebo: 3 (1 drowning, 2 burns)). The infant mortality rate was 47 per 1000 person-years-at-risk (PYR) [11]; and declined to 14 and 12 per 1000 PYR in the second and third year of life.

### Baseline characteristics

There were no baseline differences between the children who had received neonatal VAS or placebo, continued to live in the study area and were met at follow-up (Table 1). There were no differences between the children met at follow-up (groups A and B) and those not met (parts of groups C and D) with regard to treatment, sex or anthropometric measurements at enrolment. Children living in the area at 1 year of age and met at follow-up were more likely to have mothers with some schooling, to belong to the ethnic group Pepel, and to live in households with electricity, but the distribution did not differ between the neonatal vitamin A and placebo groups.

**Table 1:** Baseline characteristics of all enrolled and children who received vitamin A supplementation at the 12-month follow-up visit.

	All enrolled		Registered in the area at 1 year		Received VAS at FU visit	
	Vitamin A	Placebo	Vitamin A (A+C)	Placebo (B+D)	Vitamin A (A)	Placebo (B)
	N (%)	N (%)	N (%)	N (%)	N (%)	N (%)
Number of children	2145 (49)	2200 (51)	1787 (49)	1859 (51)	1440 (49)	1518 (51)
Boys^1^	1075 (50)	1125 (51)	893 (50)	944 (51)	722 (50)	776 (51)
Maternal schooling^2^						
No	624 (29)	638 (29)	513 (29)	541 (29)	384 (27)	415 (27)
Yes	1290 (60)	1362 (62)	1134 (63)	1186 (64)	962 (67)	1005 (66)
Maternal ethnicity^2^						
Pepel	605 (28)	606 (28)	548 (31)	545 (29)	469 (33)	465 (31)
Other	1458 (68)	1536 (70)	1219 (68)	1300 (70)	958(67)	1046 (69)
Electricity^2^						
No electricity	1379 (64)	1416 (64)	1171 (66)	1219 (66)	925 (64)	992 (65)
Electricity available	686 (32)	726 (33)	600 (34)	624 (34)	505(35)	518 (34)
Enrolment in rainy season^1^	1047 (49)	1107 (50)	862 (48)	955 (52)	714 (50)	785 (52)
Enrolment at National Hospital^1^	1220 (56)	1266 (58)	1000 (56)	1064 (57)	815 (56)	883 (58)
Registered VAS given in campaign before follow-up visit					820 (57)	861 (57)
Hospitalized before follow-up visit^2^						
Yes					99 (7)	114 (8)
No					1337 (93)	1400 (92)
Median age at follow up in months (10–90%)					15 (12–24)	15 (12–25)
Mean MUAC at follow-up visit in mm (SD)					148 (13)	148 (12)

1 Variables with two levels (e.g. included at national hospital/elsewhere) are presented by one of the levels if there is full information on all participants.

2 Numbers do not add up due to a few having missing information.

### Primary analysis: The effect of neonatal VAS on mortality after FU-VAS

**Figure 2:**
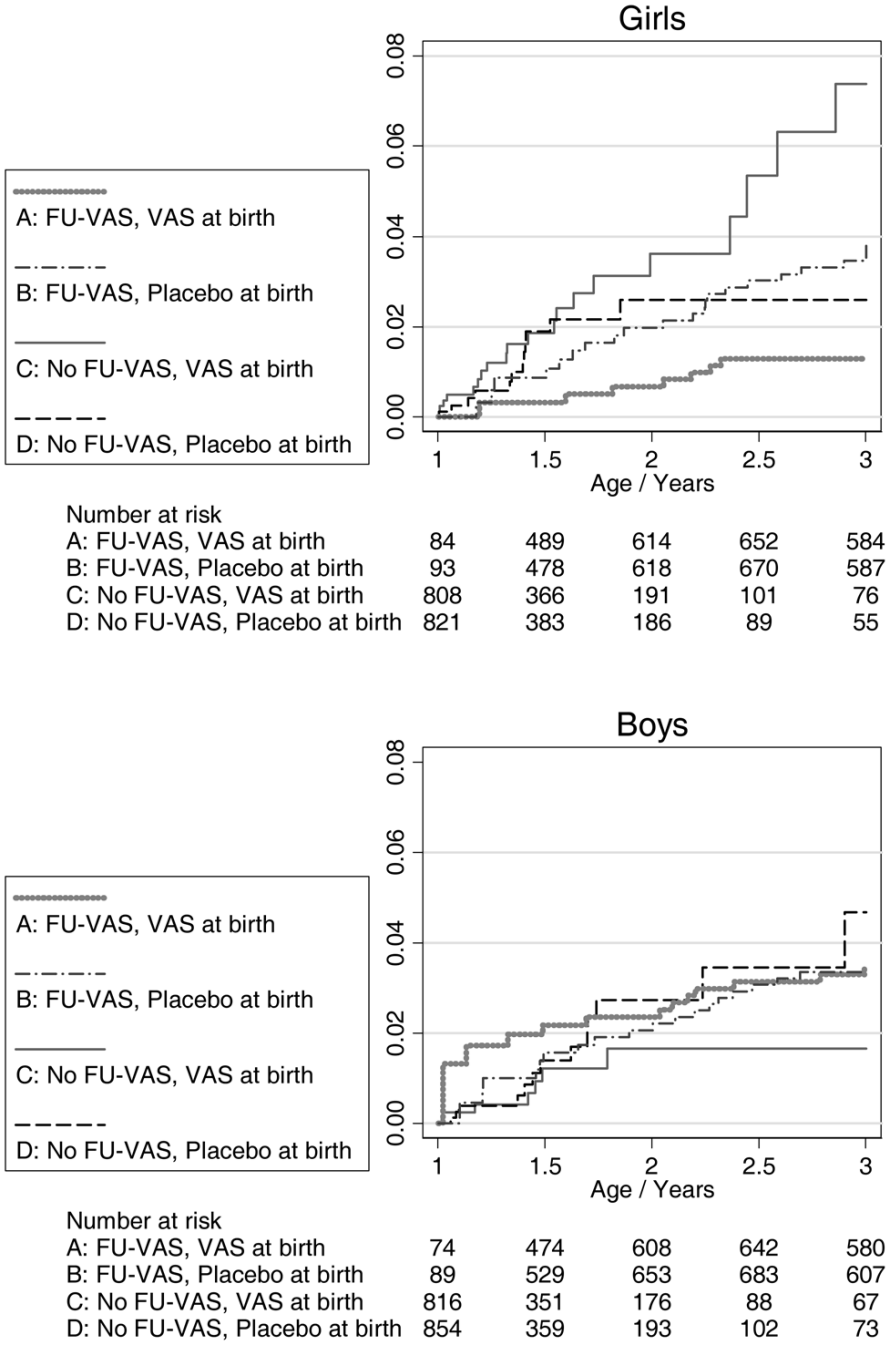
Cumulative mortality in the second and third year of life. Cumulative mortality depending on whether the children had received neonatal vitamin A (50,000 IU) or placebo and vitamin A (100,000 IU) at follow-up after 1 year of age (FU-VAS). Graphs by sex.

After FU-VAS, mortality was significantly lower for children who had received neonatal VAS (group A) than for children who had received placebo (group B) (MRR: 0.54 (0.31–0.94)) (Table 2, Figure 2). This was due to a marked effect in girls (MRR: 0.37 (0.16–0.89)), whereas there was no statistical significant effect of neonatal VAS versus placebo on survival in boys after FU-VAS: MRR: 0.73 (0.35–1.51).

**Table 2:** The effect of neonatal vitamin A/placebo on mortality in the 1^st^ 1 and 2^nd^/3^rd^ 2 year of life depending on whether the children had received neonatal vitamin A (50,000 IU) or placebo and vitamin A (100,000 IU) at follow-up after 1 year of age.

Age group	Neonatal VAS Mortality per 1000 PYRS (deaths/PYRS^3^)	Neonatal Placebo Mortality per 1000 PYRS (deaths/PYRS^3^)	Mortality Rate Ratio (VAS/placebo)	Ratio of effect of neonatal VAS after FU-VAS vs. effect of neonatal VAS after No FU-VAS	P for homogeneity of effect of neonatal VAS in the strata FU-VAS and No FU-VAS
**All children**					
0–11 months	48.7 (91/1870)	44.9 (88/1960)	1.08 (0.80–1.45)		
12–35 months					
FU-VAS^5^	8.9 (19/2145)	16.5 (37/2245)	0.54 (0.31–0.94)		
No FU-VAS^6^	26.6 (27/1016)	24.0 (25/1043)	1.10 (0.64–1.90)	0.48 (0.22–1.05)	0.07
**Boys**					
0–11 months	43.8 (41/936)	49.9 (50/1001)	0.87 (0.57–1.31)		
12–35 months					
FU-VAS^4^^,^ 5	11.3 (12/1062)	15.7 (18/1147)	0.73 (0.35–1.51)		
No FU-VAS^4^^,^ 6	14.1 (7/496)	24.6 (13/527)	0.57 (0.23–1.42)	1.28 (0.40–4.15)	0.68
**Girls**					
0–11 months	53.5 (50/934)	39.6 (38/959)	1.35 (0.89–2.06)		
12–35 months					
FU-VAS^4^^,^ 5	6.5 (7/1084)	17.3 (19/1098)	0.37 (0.16–0.89)		
No FU-VAS^4^^,^ 6	38.5 (20/520)	23.3 (12/515)	1.67 (0.81–3.42)	0.22 (0.07–0.69)	0.009

Stratified by sex.

1 Minor deviations from the previously reported estimate (11) as risk time was not censored at time of vitamin A supplementation in a campaign between 6 and 11 months.

2 Four deaths due to accidents not included.

3 PYRS = Person-years (of observation).

4 The effect of neonatal VAS differed for boys and girls depending on whether they have received FU-VAS or not; p for interaction between sex, neonatal VAS and FU-VAS: 0.035.

5 Groups A and B.

6 Groups C and D.

### Secondary analyses: Comparing the effect of neonatal VAS versus placebo after FU-VAS with the effect of neonatal VAS versus placebo before 12 months of age and the effect of neonatal VAS versus placebo in children who had not received FU-VAS

The effect of neonatal VAS versus placebo during the first year of life, prior to FU-VAS, was different from the effect after FU-VAS for girls but not for boys (Table 2). Neonatal VAS versus placebo had no significant effect on mortality in the second and third year of life in children who had received no FU-VAS (group C versus group D). In girls who had received no FU-VAS the MRR was 1.67 (0.81–3.41) and in boys it was 0.57 (0.23–1.42) similar to the estimates in the first year of life.

Hence, the effect of neonatal VAS versus placebo was significantly different in girls who had received FU-VAS (group A versus group B) and girls who had not yet received FU-VAS (group C versus group D) (ratio of effects: 0.22 (0.07–0.69), p = 0.01 for homogeneity) (Table 2). In contrast, the effect of neonatal VAS versus placebo did not differ for boys (ratio of effects: 1.28 (0.40–4.15)) (Table 2). Thus the priming effect during the second and third year of life was only seen among girls and was statistically significant from the effect among boys (p = 0.04 for interaction between sex, neonatal VAS and FU-VAS).

Fifty-seven percent of the children who received FU-VAS had received VAS in a campaign between enrolment at birth and the follow-up-visit. The participation rate was the same for boys in the VAS (58%) and the placebo (58%) group and girls in the VAS (55%) and the placebo (55%) group (p for same rate of participation = 0.43) (Table 3). After FU-VAS, children who had also received campaign VAS tended to benefit more from having received neonatal VAS compared with children who had not received VAS in a campaign. This was due to a differential effect in girls; girls who had received both campaign VAS and FU-VAS had a strong beneficial effect of neonatal VAS (MRR = 0.15 (0.03–0.67)) whereas girls who had not received campaign VAS prior to FU-VAS had no significant effect of neonatal VAS (MRR = 0.87 (0.27–2.86)) (test for interaction, p = 0.07). There was no difference for boys.

**Table 3:** The effect of neonatal vitamin A versus placebo on mortality following 100,000 IU of vitamin A given in the 2nd/3rd year of life stratified by vitamin A received in campaign before FU-VAS^1^.

Potential effect modifier	Neonatal VAS Mortality per 1000 PYRS (deaths/PYRS^2^)	Neonatal Placebo Mortality per 1000 PYRS (deaths/PYRS^2^)	Mortality Rate Ratio (Neonatal VAS/placebo)	P for homogeneity of effect of neonatal VAS in the strata Campaign VAS and No Campaign VAS
Vitamin A received in Campaign before FU-VAS				
All				
Campaign VAS	6.8 (8/1181)	17.9 (22/1228)	0.38 (0.17–0.85)	
No Campaign VAS	11.4 (11/965)	14.8 (15/1016)	0.78 (0.36–1.69)	0.21
Boys				
Campaign VAS	10.0 (6/598)	13.7 (9/658)	0.74 (0.26–2.08)	
No Campaign VAS	12.9 (6/463)	18.4 (9/489)	0.71 (0.25–2.00)	0.97
Girls				
Campaign VAS	3.4 (2/582)	22.8 (13/570)	0.15 (0.03–0.67)	
No Campaign VAS	10.0 (5/502)	11.4 (6/528)	0.87 (0.27–2.86)	0.07

1 Four deaths due to accidents not included.

2 PYRS = Person-years (of observation).

### Subgroup analyses

The effect of neonatal VAS versus placebo on survival after FU-VAS did not depend on timing of follow-up visit (data not shown), maternal education (p for same effect in children who received neonatal VAS versus placebo = 0.40/p = 0.97 for boys/girls), place of enrolment (health centre vs. hospital: p = 0.76/p = 0.22) or mid upper arm circumference (MUAC) (lowest quartile vs. other: p = 0.64/p = 0.63).

### Overall effect of neonatal VAS

Since the children were followed to 3 years of age, we measured the long-term effect of neonatal VAS versus placebo between birth and 3 years of age regardless of subsequent doses of VAS. The effect tended to be beneficial for boys, the MRR being 0.79 (0.56–1.10). In spite of the beneficial effect of boosting with FU-VAS for girls, the overall effect of neonatal VAS tended to be slightly negative for girls, the MRR being 1.14 (0.82–1.58). In the combined analysis for boys and girls, the MRR was 0.95 (0.76–1.20).

## Discussion

### Main observations

Long-term follow-up in our randomized trial of neonatal VAS versus placebo showed that children who received VAS after 12 months of age (FU-VAS) had significantly lower mortality if they had received neonatal VAS and not placebo, due to a beneficial effect in girls. The beneficial effect of neonatal VAS may have been particularly strong for girls who received several doses of subsequent VAS (campaign VAS, FU-VAS). This is in strong contrast to the lack of effect or maybe even negative effect of neonatal VAS versus placebo on infant mortality [11], an effect which continued in the second and third year of life in girls who did not receive FU-VAS. Neonatal VAS had no strong effect on survival in boys who received FU-VAS. Taken together these data suggest that priming with neonatal VAS was important, but only among girls.

### Strengths and weaknesses

Ideally children should have been randomized to VAS or placebo at birth and at 12 months to get groups A, B, C and D. This was not the case as the study was not planned. All non-randomized comparisons, i.e. comparisons between groups A and B on one hand and groups C and D on the other hand, should be interpreted with caution. However, it should be noted that the main comparison of group A versus group B as well as the comparison of group C versus group D, represent comparisons of subgroups within randomized groups and as discussed below there is no indication that the generation of these subgroups introduced bias. As hypothesized group A had lower mortality than group B, but only for girls. It could be speculated that the negative effect of neonatal VAS for infant girls [11] left only the healthier girls likely to survive in the second and third year of life. However, there was no difference in MUAC or previous hospitalizations between VAS and placebo girls at follow-up. Importantly, we also examined the mortality of recipients of neonatal VAS and placebo during the second and third year of life among those who had not yet received FU-VAS (groups C and D). Since the effects were totally different, the lower mortality of group A versus group B was not due to neonatal VAS *per se*.

Though groups C and D were the result of selection bias (not being home to receive FU-VAS) rather than initial randomization, it seems unlikely that the comparison of C versus D, and the contrast with the comparison of A versus B has been seriously biased. C versus D essentially continued the effect of neonatal VAS versus placebo on infant mortality for both boys and girls. Therefore the lower mortality of A compared with B among girls is likely to be a priming effect of neonatal VAS. This priming effect may be strongest after several doses of VAS since the beneficial effect was most pronounced in girls who had also received campaign VAS before receiving FU-VAS (Table 3).

### Consistency with other studies

The effect of repeated dosing on mortality has not been studied thoroughly. However, the first trials of VAS may suggest a similar positive effect after subsequent doses of VAS. In Ghana, the mortality ratio after the first dose of VAS versus placebo [9] was 0.99 (0.78–1.67) but declined to 0.68 (0.55–0.82) after subsequent doses. In Nepal, the mortality ratio was 0.76 (0.50–1.15) in the first 4 months of the study and 0.68 (0.45–1.00) and 0.67 (0.45–0.99) in the subsequent rounds [8]. No study has examined possible differential effect for boys and girls in detail. In India, two supplements separated by six months were given to children aged 1–5 years. The study reports no statistically significant difference between the vitamin A and placebo group after 0, 1 or 2 doses of VAS/placebo, but it appears that the female/male mortality ratio changed depending on the number of doses [7]. The pattern is compatible with a particular beneficial effect of the subsequent doses of VAS in girls as observed in the present trial. A more beneficial effect for girls than boys was also seen in a study of weekly low-dose supplements from India [17]. These studies seem to support a priming effect in other settings as well.

### Potential biological mechanisms

We can only speculate about possible mechanisms, but the explanation may lie in priming of the naïve immune system. Binding of retinoic acid, the active metabolite of retinol, to its receptors has been shown to alter interactions with proteins that induce epigenetic changes [18]. Hence, a high dose of vitamin A in early life may cause epigenetic alterations, which could lead to immediate effects on the immune system, but also prime the response to subsequent high doses of VAS. Also, vitamin A has been shown to have an up-regulating effect on the dendritic cells in the gut, enhancing their capacity to generate retinoic acid from vitamin A via positive feed-back loop [19]. Hence, if the enzymatic apparatus is stimulated by high doses of VAS in early life, this may alter the processing of subsequent doses of VAS. We have no explanation for the observed sex-differences, but sex-differential imprinting effects of neonatal VAS have been shown in rats; VAS affected later hormone content in immune cells (monocytes-macrophages-granulocytes and T-lymphocytes) in a way that differs for males and females [20].

### Implications and conclusions

Long-term follow-up in this randomized trial suggests that neonatal VAS compared with placebo primed the response to subsequent VAS in girls. If this is true, the dosing regime may influence the effect of VAS on child mortality. It should be noted that for girls neonatal VAS had a negative effect on survival until 3 years of age if no FU-VAS was given. Though we observed a beneficial effect of re-exposure to VAS in the second and third year of life this was not enough to reverse the trend towards a negative effect seen in infancy. First and foremost, it is essential to study if a beneficial effect of priming in girls can be achieved without a negative effect of the first dose; for example, is there beneficial priming when the first dose is given after 6 months of age as is currently recommended?

Our studies and the trials documenting a particularly beneficial effect of the second dose of VAS were designed to study the overall effect of VAS on mortality rather than a priming effect. However, the consistent pattern suggests that there are reasons to conduct additional randomized trials to determine the optimal interval between doses and the optimal timing of the first dose.
